# Mass Cytometry Reveals Distinct Platelet Subtypes in Healthy Subjects and Novel Alterations in Surface Glycoproteins in Glanzmann Thrombasthenia

**DOI:** 10.1038/s41598-018-28211-5

**Published:** 2018-07-09

**Authors:** Thomas A. Blair, Alan D. Michelson, Andrew L. Frelinger

**Affiliations:** Center for Platelet Research Studies, Dana-Farber/Boston Children’s Cancer and Blood Disorders Center, Harvard Medical School, Boston, MA USA

## Abstract

Mass cytometry (MC) uses mass spectrometry to simultaneously detect multiple metal-conjugated antibodies on single cells, thereby enabling the detailed study of cellular function. Here, for the first time, we applied MC to the analysis of platelets. We developed a panel of 14 platelet-specific metal-tagged antibodies (targeting cluster of differentiation [CD] 9, CD29, CD31, CD36, CD41, CD42a, CD42b, CD61, CD62P, CD63, CD107a, CD154, glycoprotein [GP] VI and activated integrin αIIbβ3) and compared this panel with two fluorescence flow cytometry (FFC) panels (CD41, CD42b, and CD61; or CD42b, CD62P, and activated integrin αIIbβ3) in the evaluation of activation-dependent changes in glycoprotein expression on healthy subject and Glanzmann thrombasthenia (GT) platelets. High-dimensional analysis of surface markers detected by MC identified previously unappreciated subpopulations of platelets in healthy donors. As expected, MC and FFC revealed that GT platelets had significantly reduced CD41, CD61, and activated integrin αIIbβ3 surface expression. MC also revealed that surface expression of CD9, CD42a and CD63 were elevated, CD31, CD154 and GPVI were reduced and CD29, CD36, CD42b, CD62P and CD107a were similar on GT platelets compared to healthy donor platelets. In summary, MC revealed distinct platelet subtypes in healthy subjects and novel alterations in surface glycoproteins on GT platelets.

## Introduction

Hemostasis is a dynamic process driven by regulated events that culminate in the arrest of bleeding^1^. Specialized surface receptors are at the forefront of this process contributing to platelet adhesion, activation, and aggregation^2,3^. Quiescent platelets express a large number of surface proteins, including CD9 (tetraspanin), CD29 (integrin β1), CD31 (platelet endothelial cell adhesion molecule [PECAM-1]), CD36 (GPIV), CD41 (integrin αIIb), CD42a (GPIX), CD42b (GPIbα), CD61 (integrin β3) and GPVI^2,3^. Surface levels of some of these molecules (*e*.*g*., CD41 and CD61) are increased following platelet activation, while other proteins which are virtually absent from the surface of resting platelets, such as CD62P (P-selectin), CD63 (lysosome-associated membrane protein [LAMP]-3), CD107a (LAMP-1) and CD154 (CD40 ligand), are translocated from intracellular stores to the plasma membrane following activation^2,3^. Differences in expression levels of these surface receptors, before and after platelet activation, has been suggested to contribute to response heterogeneity in thrombus formation^4,5^. Intrinsic differences in platelet surface receptor density present at the time platelets are formed from megakaryocytes, which themselves may be heterogenous^6^, and changes due to platelet ageing and/or activation history have been suggested to contribute to the heterogeneous nature of circulating platelets^5^. Whether these variations correspond to distinct platelet subpopulations with specialized functions, similar to the dedicated roles of subsets of immune cells, remains unknown. A defect in any one of these key receptors on platelets can lead to a bleeding disorder. For example, Glanzmann thrombasthenia (GT), an autosomal recessive inherited disorder characterized by a quantitative and/or qualitative defect in the platelet fibrinogen receptor, integrin αIIbβ3 (CD41/CD61) causes increased bleeding in patients^7,8^.

Flow cytometric analysis of platelets stained with receptor-specific monoclonal antibodies conjugated to fluorescent probes (fluorescence flow cytometry or FFC) is traditionally used in clinical and research settings to study platelet function and to diagnose patients with inherited platelet disorders^9,10^. FFC, in contrast to nearly all other platelet function tests, allows evaluation of platelet function in small blood volumes and in samples with reduced platelet counts because this method tests the function of individual platelets (albeit very rapidly; ~10,000 platelets/min)^10,11^. FFC has also been an effective tool to monitor the effects of antiplatelet agents on platelet function in human clinical trials and in animal models^12–18^. However, a major drawback of FFC is that the number of parameters that can be simultaneously analyzed is inherently limited by emission spectra overlap^19^. Increasing the number of fluorophores present within an FFC antibody panel increases the likelihood of spectral overlap and the complexity of the compensation required for accurate analysis. Consequently, platelet-specific FFC antibody panels typically consist of no more than 3 markers^9,20,21^.

Mass cytometry (MC) is a next generation flow cytometry platform that enables simultaneous phenotypic and functional analysis of multiple parameters on individual cells. MC employs probes (*e*.*g*., antibodies, lectins, RNA probes, intercalators) that are conjugated to heavy metal isotopes, flow cytometric analysis of single-cells like FFC, and time-of-flight mass spectrometry as a detection technique^22,23^. The platform overcomes many of the limitations associated with FFC and offers several major advantages over conventional fluorescent-based applications. The detection of unique isotope masses which have minimal spectral or channel overlap eliminates the need for compensation, resulting in an order of magnitude increase in the number of cellular parameters that can be analyzed simultaneously on individual cells. MC panels of up to 45 different cellular parameters on peripheral blood mononuclear cells have been reported^24^. However, the MC platform is theoretically capable of simultaneously measuring up to 100 different parameters^25,26^. Expanding the number of parameters that can be simultaneously monitored in a single tube guarantees that staining conditions are identical for all parameters, thereby minimizing pre-analytical variables. Unlike FFC, MC does not allow the detection of forward- or side-scatter light properties of cells and consequently cannot be used to determine cell size or granularity. However, novel labeling methods to characterize cell size by MC using wheat germ agglutinin or osmium tetroxide have recently been reported^27^.

MC has not previously been used in the analysis of platelet surface antigens. In this study, we have developed and validated a novel platelet-specific metal-tagged antibody panel that enables the simultaneous detection of 14 different surface antigens (CD9, CD29, CD31, CD36, CD41, CD42a, CD42b, CD61, CD62P, CD63, CD107a, CD154, GPVI and activated integrin αIIbβ3) by MC. We used this panel to directly compare MC with FFC for the evaluation of activation-dependent changes in cell-surface antigen expression on platelets from healthy donors and GT patients. We found that (i) high-dimensional analysis of surface antigens detected by MC reveals novel platelet subpopulations in healthy subjects and (ii) MC identifies previously unappreciated alterations in surface glycoprotein expression on GT platelets.

## Results

### Comparing MC and FFC for the evaluation of agonist-induced integrin αIIbβ3 activation (PAC1) and P-selectin expression (CD62P)

**Figure 1:**
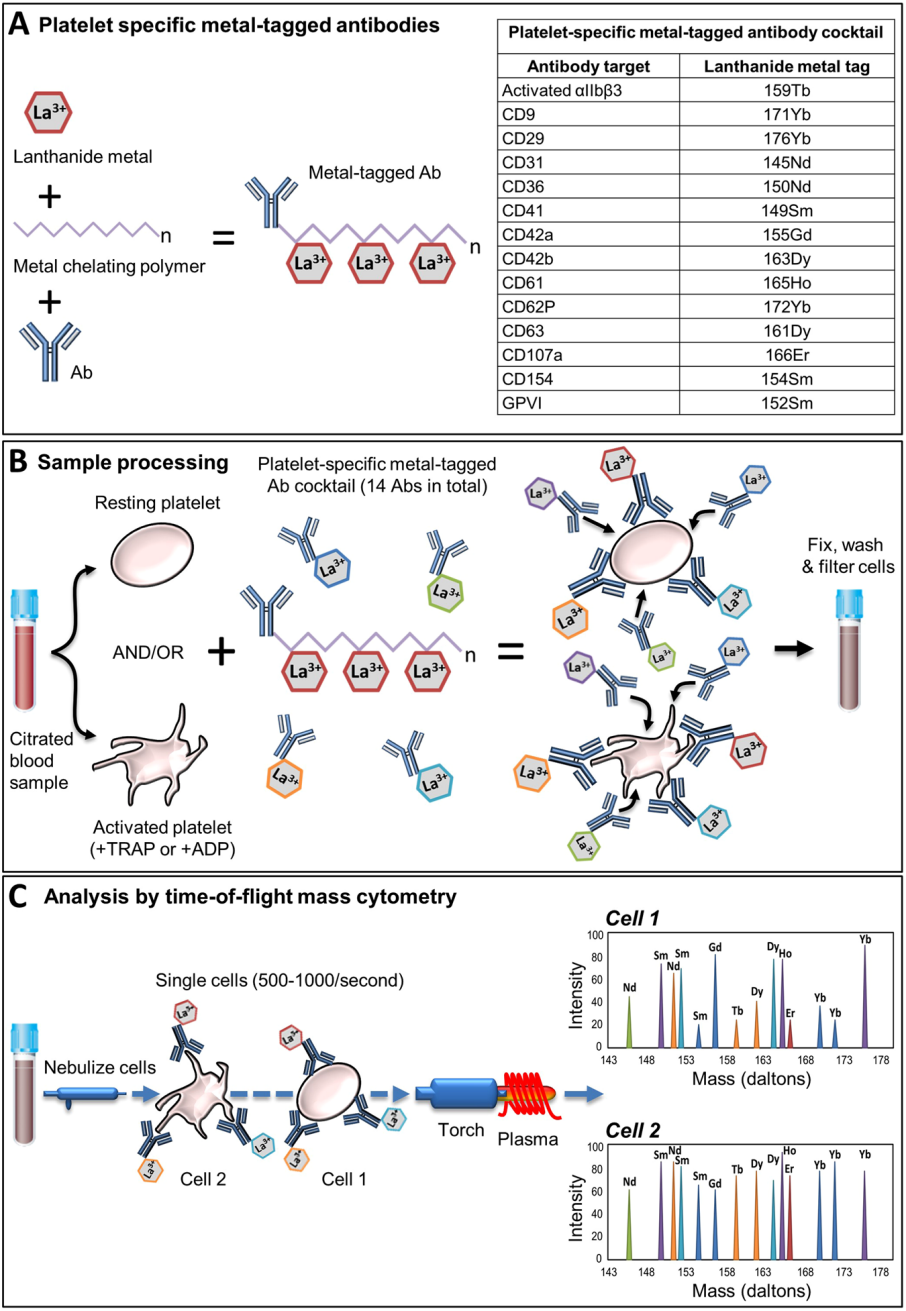
Schematic overview of time-of-flight MC for simultaneous analysis of multiple platelet surface markers. **(A)** A platelet-specific panel of metal-tagged antibodies targeting surface antigens of interest was constructed. Each antibody is bound to 2–4 chelating polymers that are attached to stable lanthanide metal isotopes. Each polymer contains approximately 25–30 lanthanide ions of the same mass. **(B)** Platelets from a patient blood sample were incubated with the platelet-specific metal-tagged antibody cocktail under stimulating or non-stimulating conditions. Samples were fixed with 1% formaldehyde, washed with ddH_2_0 to remove salts and filtered through a 35 µm cell strainer. **(C)** Samples were then analyzed using time-of-flight inductively coupled plasma MC. Samples were nebulized into single-cell droplets and passed through a 7500 K argon plasma where they were vaporized, atomized and ionized to form clouds of ions that correspond to individual cells. Each ion within the cloud was detected and separated according to mass and correlated with a specific metal-tagged probe present in the antibody cocktail. Abbreviations: Ab, antibody; ADP, adenosine diphosphate; CD, cluster of differentiation; GP, glycoprotein; La, Lanthanide; TRAP, thrombin receptor activating peptide.

To compare the MC and FFC platforms for platelet analysis we first designed a novel metal-tagged MC antibody panel to target well-established surface markers on platelets (Fig. 1A, Supplemental Table S1) including platelet surface P-selectin (monitored with anti-CD62P-172Yb) and platelet surface activated integrin αIIbβ3 (monitored with the activation-dependent monoclonal antibody PAC1, labeled in-house with 159Tb). The specificity of PAC1-159Tb for activated αIIbβ3 was assessed by its dependence on platelet activation for binding and by its blockade by the αIIbβ3 inhibitor, eptifibatide (Supplemental Figure S1A-B). Platelet surface activated αIIbβ3 expression in whole blood stimulated with TRAP/ADP (200 µM) was, as expected, significantly elevated compared with unstimulated controls (Supplemental Figure S1A-B). Inclusion of eptifibatide (2.5 µg/mL) in the reaction mixture completely blocked anti-PAC1-159Tb binding to activated integrin αIIbβ3 following TRAP/ADP (200 µM) stimulation, thus confirming the specificity of anti-PAC1-159Tb for its antigen (Supplemental Figure S1A,B).

**Figure 2:**
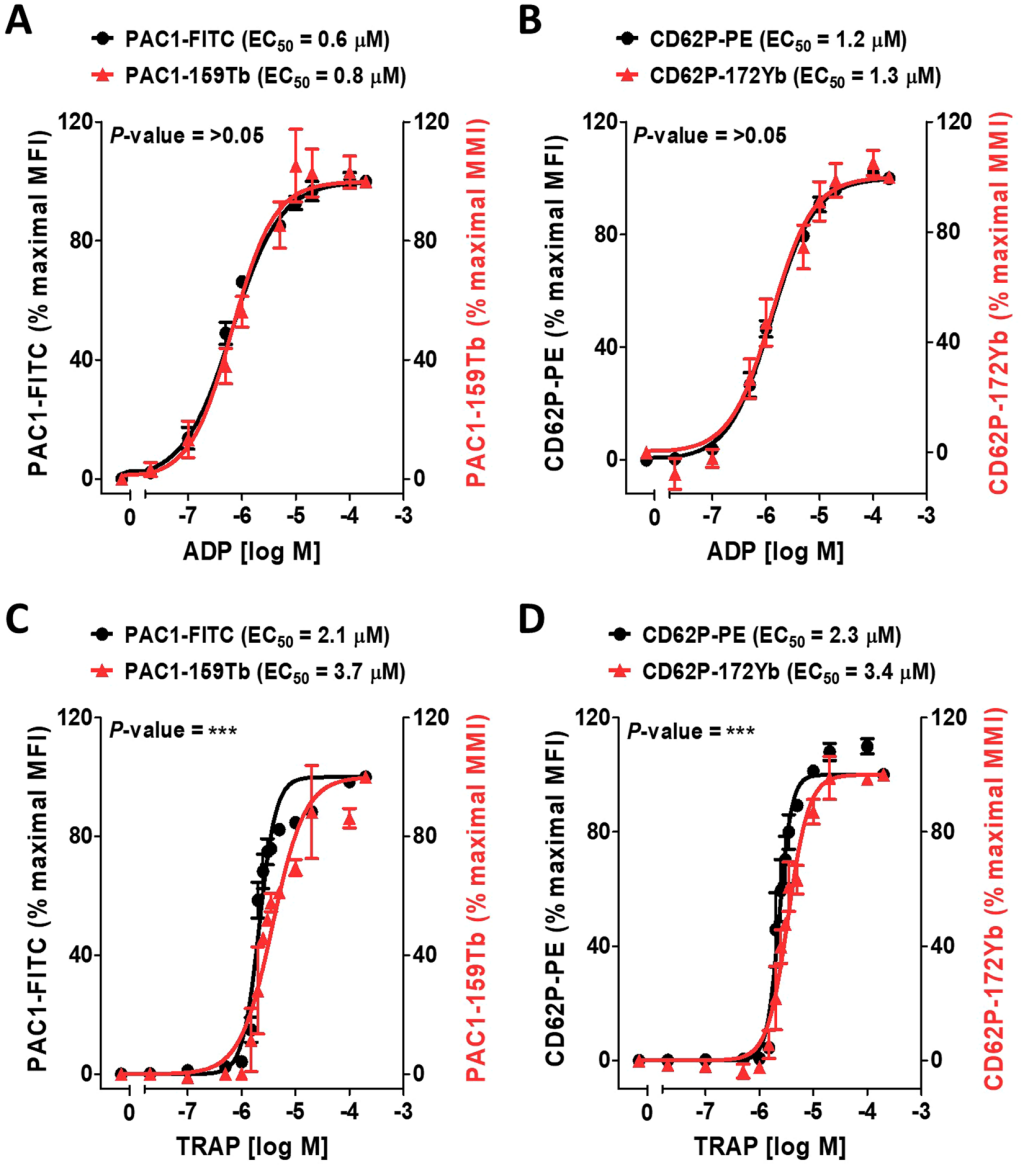
Comparing MC and FFC platforms for measurement of agonist-stimulated integrin αIIbβ3 activation (PAC1) and P-selectin expression (CD62P) on platelets. Citrate-anticoagulated blood from 3 separate healthy donors was treated with vehicle or the indicated concentrations of ADP (**A**,**B**) or TRAP (**C**,**D**); for 30 minutes in the presence of PAC1-FITC or PAC1-159Tb antibodies to assess integrin αIIbβ3 activation (**A**,**C**) and CD62P-PE or CD62P-172Yb antibodies to assess α-granule secretion (**B**,**D**). Samples were fixed in 1% formaldehyde and analyzed by MC or FFC. Data were analyzed using a non-linear fit of log agonist *vs*. response; variable slope in GraphPad Prism 5. Results are expressed as a percentage of mean metal intensity (MMI; mass cytometry readout) or the mean fluorescence intensity (MFI; flow cytometry readout) achieved with 200 µM ADP or TRAP, respectively (means ± SEM; n = 2/3, with n = 2 accounting for the linear region of the TRAP dose-response [concentrations 1.5–3.5 µM] and n = 3 accounting for all other concentrations [0–1 µM & 5–200 µM]). Statistical analysis: an extra sum-of-squares F test was used to determine whether the EC_50_ values of the curves differed significantly; ***P < 0.001. Abbreviations: ADP, adenosine diphosphate; FFC, fluorescence flow cytometry; FITC, fluorescein isothiocyanate; MC, mass cytometry; PE, phycoerythrin; TRAP, thrombin receptor activating peptide.

We next compared MC and FFC platforms for evaluating platelet activation by incubating platelets with CD62P-172Yb and PAC1-159Tb antibodies or with their fluorescent antibody counterparts, CD62P-PE and PAC1-FITC with and without various concentrations of TRAP or ADP. Agonist-induced increases in platelet surface activated αIIbβ3 and P-selectin using MC and FFC platforms were similar and the results were highly correlated (R^2^ ≥ 0.9, Fig. 2). The EC_50_ values for ADP-induced platelet surface activated αIIbβ3 measured with anti-PAC1-159Tb and anti-PAC1-FITC were not significantly different (EC_50_s 0.8 µM and 0.6 µM by MC and FFC respectively, *P*-value = 0.07, Fig. 2A). Similarly, EC_50_ values for ADP-induced platelet surface P-selectin expression measured with CD62P-172Yb and CD62P-PE were not significantly different (EC_50_s 1.3 µM and 1.2 µM by MC and FFC respectively, *P*-value = 0.74, Fig. 2B). The EC_50_ values obtained for TRAP-induced activation of αIIbβ3 by MC differed slightly but significantly from that determined by FFC (EC_50_s 3.7 µM *vs*. 2.1 µM by MC *vs*. FFC respectively, *P* < 0.001, Fig. 2C). Similarly, the EC_50_s for TRAP-induced platelet surface P-selectin expression by MC *vs*. FFC differed slightly but significantly (EC_50_s 3.4 µM *vs*. 2.3 µM by MC *vs*. FFC respectively, *P* < 0.001, Fig. 2D).

### MC enables an order of magnitude more cellular parameters than FFC to be assessed simultaneously during platelet activation

**Figure 3:**
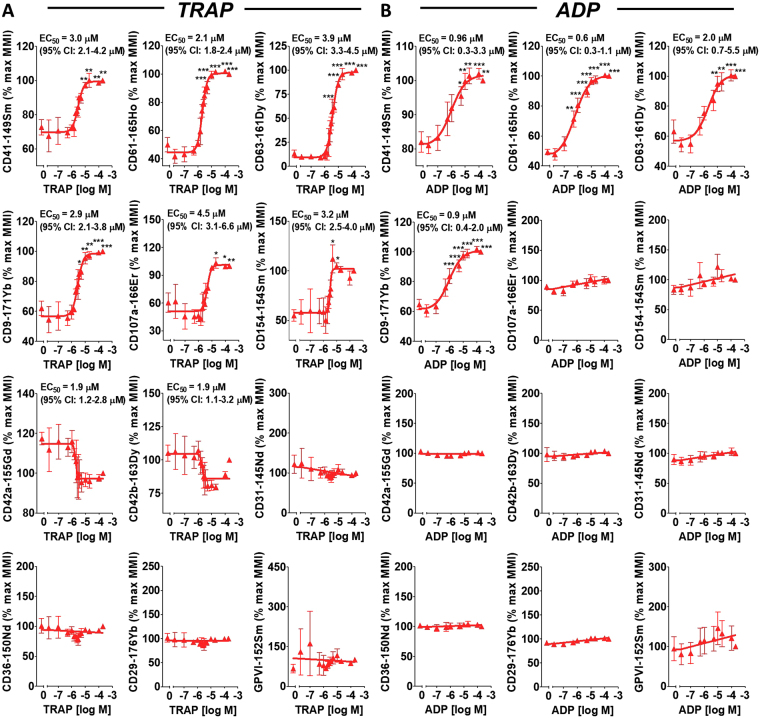
MC enables an order of magnitude more parameters than FFC to be analyzed simultaneously during platelet activation. Citrate-anticoagulated blood from the same 3 separate healthy donors in Fig. 2 was simultaneously treated with vehicle or the indicated concentrations of TRAP (**A**) or ADP (**B**) for 30 minutes in the presence of a custom platelet-specific, metal-tagged antibody panel. This panel contained antibodies directed against CD41, CD61, CD63, CD9, CD107a, CD154, CD42a, CD42b, CD31, CD36, CD29 and GPVI. Samples were fixed in 1% formaldehyde and analyzed by MC. Data were analyzed using a non-linear fit of log agonist *vs*. response; variable slope or linear regression in GraphPad Prism 5. Results are expressed as a percentage of the mean metal intensity (MMI) achieved with 200 µM ADP or TRAP (means ± SEM; n = 2/3, with n = 2 accounting for the linear region of the TRAP dose-response [concentrations 1.5–3.5 µM)] and n = 3 accounting for all other concentrations [0–1 µM and 5–200 µM]). Statistical analysis: 1-way ANOVA was used in conjunction with a Dunnett multiple comparison test (all results compared to vehicle control) to indicate statistical significance; *P < 0.05, **P < 0.01 and ***P < 0.001. Abbreviations: ADP, adenosine diphosphate; FFC, fluorescence flow cytometry; MC, mass cytometry; TRAP, thrombin receptor activating peptide.

Figure 3 shows the results, obtained in parallel with PAC1-159Tb and CD62P-172Yb (Fig. 2), for the 12-additional metal-tagged antibodies present in the MC panel. MC revealed that platelet surface CD41, CD61, CD63, CD9, CD107a and CD154 were elevated in a dose-dependent manner with TRAP and ADP stimulation (Fig. 3A,B). Platelet surface CD42a and CD42b showed a trend to be dose-dependently decreased with TRAP stimulation. Surface expression of CD42a and CD42b stayed constant over an array of ADP concentrations (Fig. 3A,B). CD31, CD36, CD29 and GPVI were constitutively expressed and surface plasma membrane levels remained constant at varying concentrations of TRAP and ADP (Fig. 3A,B).

### Identification of platelet subpopulations by high-dimensional viSNE analysis

**Figure 4:**
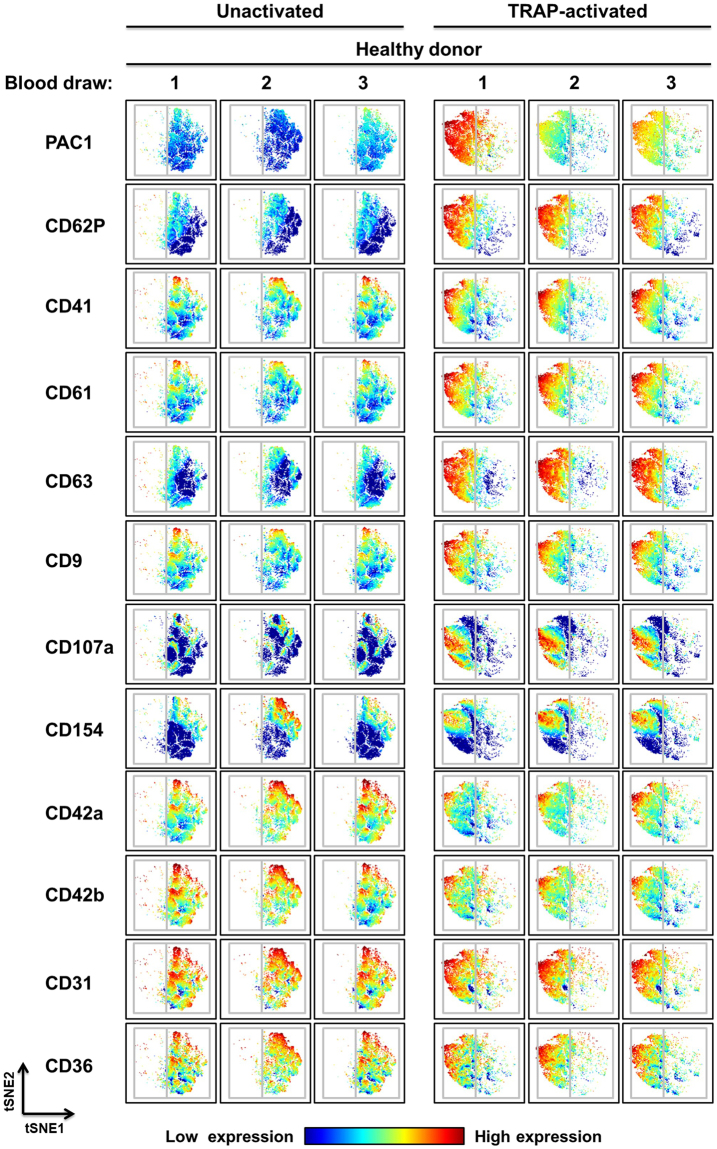
Multidimensional analysis of platelet subpopulations by MC reveals heterogeneity in healthy donor samples. Visual stochastic neighbor embedding (viSNE) plots of whole blood samples drawn on 3 separate days from the same healthy subject (a different healthy subject from the healthy subjects analyzed in Figs 2 and 3). Samples were stained with a metal-tagged antibody cocktail containing 12 markers (directed against: CD9, CD31, CD36, CD41, CD42a, CD42b, CD61, CD62P, CD63, CD107a, CD154 and activated integrin αIIbβ3), treated with vehicle or 20 µM TRAP, and analyzed using MC. Color intensity relates to antigen expression (low [blue] or high [red]) and each dot represents an individual platelet. The distance between dots/platelets and populations of dots/platelets is inversely proportional to how closely related those dots/platelets are in terms of antigen expression and characteristics. Abbreviations: TRAP, thrombin receptor activating peptide; tSNE, t-distributed stochastic neighbor embedding.

While Fig. 3 shows the convenience of using MC to rapidly evaluate changes in multiple markers, similar analyses could be done by conventional FFC, albeit with more difficulty. To take advantage of the simultaneous measurement of multiple markers on individual cells we sought to determine whether the increases in the mean metal intensity (MMI) (for all gated platelets) for CD41, CD61, CD63, CD9, CD107a and CD154 with ADP and TRAP stimulation corresponded to similar increases in these markers on all platelets or whether the increases in the MMI were driven by subsets of platelets expressing high levels of one or several markers. To accomplish this, we used viSNE analysis to visualize high-dimensional single-cell data obtained from a healthy donor across 3 separate blood donations (Fig. 4). viSNE is an unsupervised single-cell cluster analysis tool that generates an optimized 2-dimensional representation of high-dimension data based on the t-Distributed Stochastic Neighbor Embedding (tSNE) algorithm^28^. Individual platelets, each represented by a dot, are grouped together in regions on the viSNE map based on the degree of similarity of the expression patterns of all 12 parameters assessed during the experiment. Interestingly, viSNE analysis demonstrated heterogeneity in circulating platelets by identifying subpopulations of platelets with unique antigen expression profiles (Fig. 4). After activation, most platelets stained intensely for PAC1 (activated integrin αIIbβ3), CD62P and CD63 expression, yet subsets of these platelets differed with respect to CD31 (note the CD31 dim population), CD107a (CD107a bright in lower left and middle left of panel *vs*. CD107a dim in upper left of panel) and CD154 (CD154 bright in the upper left quadrant *vs*. CD154 dim in the lower left quadrant) expression (Fig. 4). Differences in CD154 staining prior to activation (CD154 bright in upper right quadrant *vs*. CD154 dim in lower right quadrant) demonstrates that heterogeneity was present in circulating platelets prior to *ex vivo* stimulation (Fig. 4). Using viSNE analysis we were also able to identify platelet subpopulations that were common between different healthy donors, as well as subpopulations that were unique to particular donors (see Supplemental Figure S2). Following TRAP-activation, there was a large subpopulation of platelets that stained intensely for CD41, CD61, CD62P, CD63, CD107a, and PAC1 in healthy donors 1 and 2 that was absent in healthy donor 3 (see Supplemental Figure S2). Following TRAP activation, there was also a very distinct subpopulation of platelets that stained intensely for CD41, CD61, CD62P, CD63, CD107a, and PAC1 in healthy donor 3 that was absent in healthy donors 1 and 2 (see Supplemental Figure S2).

### MC reveals novel alterations in the platelet surface expression of antigens in GT patients

**Figure 5:**
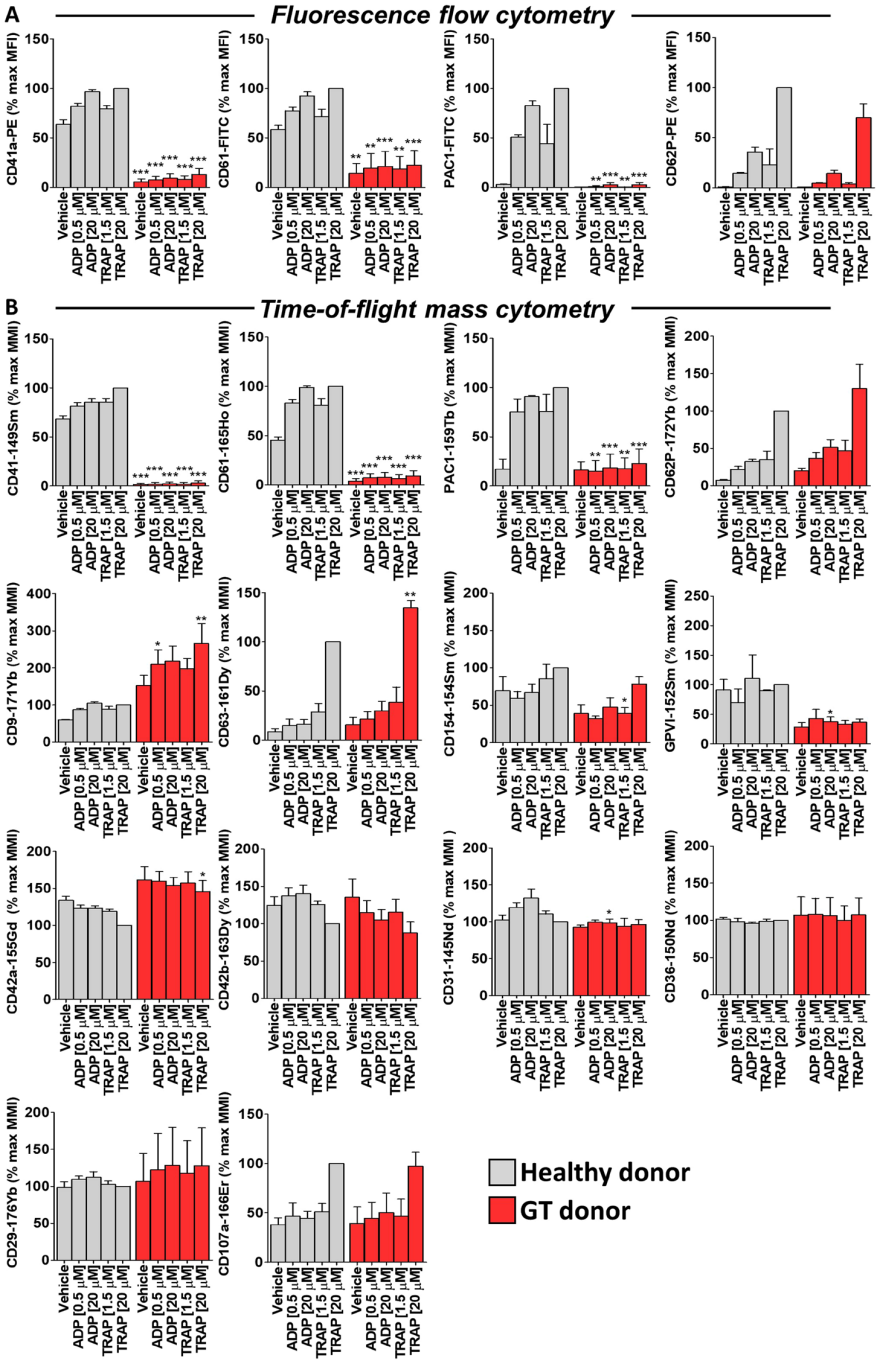
MC reveals novel alterations in the platelet surface expression of antigens in Glanzmann thrombasthenia (GT) patients. Citrate-anticoagulated blood samples from healthy donors (n = 3) and a GT patient (3 separate blood draws from the same patient on 3 different visits) were treated with vehicle, ADP (0.5 or 20 µM) or TRAP (1.5 or 20 µM) for 30 minutes in the presence of a fluorescent-tagged antibody cocktail **(A) (**CD41a-PE and CD61-FITC or PAC1-FITC and CD62P-PE) or a custom metal-tagged antibody cocktail (**B**) (CD41-149Sm, CD61-165Ho, PAC1-159Tb, CD62P-172Yb, CD63-161Dy, CD9-171Yb, CD154-154Sm, CD42a-155Gd, CD42b-163Dy, GPVI-152Sm, CD31-145Nd, CD36-150Nd, CD29-176Yb and CD107a-166Er). Samples were fixed in 1% formaldehyde and analyzed by flow cytometry or mass cytometry. Results are expressed as a percentage of the mean fluorescence intensity (MFI; flow cytometry readout) or mean metal intensity (MMI; mass cytometry readout) achieved with 20 µM TRAP in healthy donor platelets (means ± SEM; n = 3). Statistical analysis: 2-way ANOVA was used in conjunction with a Bonferroni post-test to indicate statistical significance; *P < 0.05, **P < 0.01 and ***P < 0.001. Abbreviations: ADP, adenosine diphosphate; FITC, fluorescein isothiocyanate; MC, mass cytometry; PE, phycoerythrin; TRAP, thrombin receptor activating peptide.

We used GT platelets to validate the use of MC as a research tool by comparing data obtained using MC with that obtained using FFC. Both MC and FFC analysis platforms showed, as expected, greatly reduced surface expression of CD41, CD61 and activated integrin αIIbβ3 on GT platelets, both without and with *ex vivo* stimulation (0.5 or 20 µM ADP or 1.5 or 20 µM TRAP) compared to that on healthy control platelets (Fig. 5A,B). The absence of binding of PAC1-159Tb, CD41-149Sm and CD61-165Ho to platelets genetically deficient in αIIbβ3 confirms the specificity of these reagents. Platelet surface P-selectin (CD62P) expression following stimulation with ADP (0.5 or 20 µM) or TRAP (1.5 or 20 µM) as measured by both MC and FFC platforms was similar on platelets from GT patients and non-GT controls (Fig. 5A,B). MC enabled 10 additional surface markers to be simultaneously measured revealing elevated surface level expression of CD9, CD42a and CD63, reduced levels of CD31, CD154 and GPVI, and similar levels of CD29, CD36, CD42b and CD107a on GT platelets compared to non-GT healthy control platelets (Fig. 5B).

## Discussion

Here, we report the first use of MC to evaluate platelet surface glycoproteins and function. Since its introduction 30 years ago^29^, FFC has been, the gold-standard analytical tool to measure platelet surface antigens^30^. The number of parameters simultaneously detected by FFC is, however, inherently limited by spectral overlap of fluorophore emissions. MC overcomes these limitations by employing metal-tagged antibodies and time-of-flight mass spectrometry to simultaneously analyze on individual cells an order of magnitude more platelet surface antigens than FFC. We developed a novel MC metal-tagged antibody panel for simultaneous analysis of 14 different platelet surface antigens. This panel and method were validated by (i) direct comparison against data obtained using FFC, (ii) changes in reactivity with agonist-stimulated *vs*. unstimulated platelets, (iii) inhibition with specific blocking reagents and (iv) reactivity with platelets genetically deficient in integrin αIIbβ3 (GT platelets). The optimized panel was used to study activation-dependent changes in surface antigen expression on healthy donor and GT patient platelets. MC revealed previously unappreciated subpopulations of platelets in healthy donors and novel alterations in surface glycoproteins on GT platelets.

Previous studies have tended to treat platelets as a single population. However, circulating platelets differ one from another with respect to their (a) size^31,32^, (b) surface receptor expression^33–35^, (c) glycosylation^36^, (d) granule content^37,38^, (e) response to agonist stimulation^39–41^, and (f) participation in thrombus formation^4^. The factors contributing to this variability may include heterogeneity among platelet-producing megakaryocytes^6^, differences relating to platelet age^31,36,42^, and differences in exposure to local, *in vivo* activating conditions which may lead to changes in expression of surface molecules and desensitization to further activation^43,44^. In patients with immune thrombocytopenia, increased surface P-selectin on some circulating platelets and decreased numbers of platelets that become positive for surface P-selectin and activated integrin αIIbβ3 is associated with more severe bleeding scores^45,46^. Recent studies^47^ demonstrated a subpopulation of platelets that lack endothelial nitric oxide synthase (eNOS), fail to produce nitric oxide, and have a down-regulated soluble guanylate cyclase signaling pathway. As a result, this subpopulation of platelets showed greater adhesion to collagen, activation of αIIbβ3, and formed larger aggregates than eNOS-positive platelets^47^.

Thus, ample evidence exists for variability among platelets in health and disease. However, until now, it has been difficult to determine whether variation in one platelet parameter corresponded to variation in other parameters, thereby defining distinct platelet subpopulations. This is primarily due to the inherent limitations of FFC, whereby the use of fluorescent probes restricts the number of parameters that can be simultaneously analyzed. Here we demonstrate the utility of MC and a custom platelet-specific metal-tagged antibody panel for identifying platelet subsets in healthy individuals. After activation, we found most platelets, as expected, stained intensely for activated integrin αIIbβ3, CD62P and CD63, yet a subset of these platelets differed with respect to CD31 expression (Fig. 4). Given that CD31 may be a negative modulator of platelet activation pathways^48,49^, the presence of a subset of platelets with low levels of CD31 would suggest that these platelets may be less susceptible to down-regulation. In the absence of *in vitro* agonist stimulation, a platelet subset with high CD154 (CD40 ligand) was identified (Fig. 4), suggesting prior activation and possible desensitization of these platelets. We also identified subpopulations of platelets that were common among healthy donors and subpopulations of platelets that were unique to a subset of healthy subjects (see Supplemental Figure S2). More work is required to completely map and characterize the different platelet subpopulations that exist in a large cohort of healthy donors and to gain greater insight into the factors (*e*.*g*., diet, diurnal variation, disease, age, *etc*.) that may affect these subpopulations. Like all platelet function tests, MC could be susceptible to variability in sample processing technique. However, we minimized this possibility by using the same phlebotomist, the same researcher, the same lot of antibodies and agonists, and drawing the blood at approximately the same time each day. Furthermore, the fact that platelets were exposed to all 10-12 antibodies simultaneously in the same tube further reduced the possibility of pre-analytical variables. The fact that we identified the same populations across 3 separate blood draws spanning 4 months in the same donor suggests that variables in sample processing techniques were indeed kept to a minimum. Overall, these data demonstrate MC to be a more effective tool than FFC for the complete mapping of the heterogeneity that exists within healthy donor populations and disease populations.

GT platelets were used to validate MC for assessing platelet function and to demonstrate the platform’s power as a research tool. In agreement with previous findings^7,8,50^, both MC and FFC demonstrated CD41, CD61 and activated αIIbβ3 expression to be significantly reduced on GT platelets compared to control platelets under both non-stimulating and stimulating conditions. MC enabled us to survey an array of additional surface antigens and, in agreement with previous reports^51–55^, we found CD29, CD36, CD62P, and CD107a membrane expression to be similar on GT and control platelets following agonist stimulation.

CD9 levels on GT platelets have previously been reported to be similar to levels on healthy donor platelets^56^. MC revealed significantly elevated CD9 surface expression on platelets from our GT patient cohort following agonist stimulation (0.5 µM ADP and 20 µM TRAP) compared to healthy donor platelets (Fig. 5B). In platelets, CD9 co-localizes with αIIbβ3 in α-granules and in specific microdomains on the plasma membrane^57,58^. Possible explanations for increased CD9 expression on GT platelets include, (i) increased unoccupied membrane area due to the absence of integrin αIIbβ3 allowing easier insertion of CD9 in the plasma membrane, and (ii) improved CD9 antibody access to CD9 due to reduced steric hindrance. Our CD9 observations are in contrast to findings by Cramer *et al*.^56^, who used immunogold labeling and electron microscopy to report comparable qualitative labeling patterns for CD9 in GT and healthy control platelets. These discrepancies may be a consequence of the technical limitations associated with accurate quantitation of immunogold localization^59^. Furthermore, because Cramer *et al*.^56^, analyzed a vastly smaller platelet population (15 platelet equatorial sections on average) in comparison to our MC studies (30,000–50,000 cellular events per condition), their data may not be completely representative of the whole GT platelet population. Additionally, in their studies, GT platelets were only analyzed under resting conditions and not following agonist challenge^56^.

CD63 was significantly elevated on GT platelets compared to healthy control platelets following TRAP stimulation. CD63 is found on dense granule and lysosomal membranes of resting platelets and upon activation becomes expressed on the plasma membrane, where it associates with the integrin αIIbβ3-CD9 complex and with the actin cytoskeleton via αIIbβ3^60^. Similar to CD9, CD63 expression may be limited by membrane protein crowding, and in the absence of αIIbβ3 there would be less crowding. Interestingly, studies have shown that some GT patient platelets show increased surface expression of CD63, but not CD107a or CD62P following FcγRIIA crosslinking^55^. The investigators of these studies hypothesized that increased dense granule exocytosis was responsible for the increased surface expression of CD63^55^.

We observed ADP-induced CD31 surface expression to be significantly reduced on GT platelets compared to healthy control platelets. A previous study showed no difference in CD31 in GT patients, but this study immunoblotted whole platelet lysates, thus measuring total platelet CD31 levels not platelet surface expression of CD31^61^. As expected^62,63^, CD42a and CD42b surface expression on GT and healthy control platelets were relatively comparable in the present study; although subtle, yet significant, differences in surface levels of CD42a were seen with 20 µM TRAP treatment, which may be attributable to donor-to-donor variation in surface expression patterns.

TRAP- and ADP-induced dose-dependent increases in integrin αIIbβ3 activation and P-selectin expression, as determined by MMI or MFI, were highly correlated (R^2^ = 0.9186 or 0.8995, respectively). While this finding may be largely expected, it should be noted that the monoclonal antibody used to detect activated αIIbβ3 is an IgM antibodies and the labeling of an IgM with the metal chelating polymer has not previously been reported. In fact, the manufacturer recommendation is that IgM not be labeled using this procedure^64^. Nevertheless, purified PAC1 labeled in-house with 159Tb using the identical procedure recommended for IgG antibodies^64^ demonstrated high-affinity binding to platelets, which was activation dependent and could be blocked by the integrin αIIbβ3 antagonist eptifibatide. Other technical hurdles which were overcome during our development of the MC procedure for platelets include optimization of sample volume, sample type (whole blood was used to avoid pre-analytical artifacts associated with isolation of platelet-rich plasma), fixative solutions and washing conditions (washing is not required for FFC but is required for MC in order to avoid exposure of the mass spectrometer to damaging salts). Platelet recovery after fixation and wash procedures was determined to be >80% (data not shown). While the present study is focused on the analysis of platelet surface markers by MC, MC can also be used to investigate intracellular markers (*e*.*g*., phospho-proteins, cytokines, chemokines, *etc*.) in other cell types^24,65,66^. Specifically, following staining of surface markers, samples are fixed, gently permeabilized, and a panel of metal-tagged antibodies to intracellular markers is added. Thus, the MC antibody panel described in the present study can be adapted in the future to study intracellular platelet signaling.

In conclusion, MC analysis of platelets as described herein reveals novel platelet subtypes in healthy subjects and previously unreported changes in platelet surface glycoproteins on GT platelets. Moreover, this methodology provides the means to investigate the role of these platelet subsets in health and disease.

## Methods

### Materials

Metal-conjugated monoclonal antibodies were from Fluidigm Corporation (San Francisco, CA): anti-CD9-171Yb (clone SN4 C33A2), anti-CD31-145Nd (clone WM59), anti-CD61-165Ho (clone VI-PL2); from Longwood Medical Area Antibody Core (Boston, MA): anti-CD36-150Nd (clone 5-271), anti-CD42b-163Dy (clone HIP1), anti-CD41-149Sm (clone HIP8), anti-CD62P-172Yb (clone AK4), anti-CD63-161Dy (clone H5C6), anti-CD107a-166Er (clone H4A3), anti-CD154-154Sm (clone 24-31); or labeled in-house (described below): anti-CD29-176Yb (Biolegend, San Diego, CA, clone TS2/16), anti-CD42a-155Gd (BD Biosciences, San Jose, CA, clone ALMA.16), anti-GPVI-152Sm (EMD Millipore, Billerica, MA, polyclonal IgG), anti-activated αIIbβ3 (PAC1)-159Tb (BD Biosciences, San Jose, CA, clone PAC1). Fluorescent-conjugated monoclonal antibodies were from BD Biosciences (San Jose, CA): anti-CD41-PE (clone HIP8), anti-CD42b-PE-Cy5 (clone HIP1), anti-CD62P-PE (clone AK4), anti-activated αIIbβ3 (PAC1)-fluorescein isothiocyanate (FITC) (clone PAC1); or Agilent (Santa Clara, CA): anti-CD61-FITC (clone Y2/51). MaxPAR X8 Antibody Labeling Kits, Iridium 191/193 Cell-ID DNA Intercalator, and EQ Four Element Calibration Beads were from Fluidigm Corporation (San Francisco, CA). Antibody Stabilization Buffer was from Candor Biosciences (GmbH, Wangen, Germany). Amicon 3 kDa (Cat# UFC500396) and 50 kDa (Cat# UFC505096) centrifugal filter units were from EMD Millipore (Burlington, MA). Bovine serum albumin, sodium azide, HEPES [N-(2-Hydroxyethyl) piperazine-N′-(2-ethanesulfonic acid)], and tris(2-carboxyethyl)phosphine (TCEP) bond breaker were from Sigma Aldrich (St. Louis, MO). Protease-activated receptor 1 (PAR1) thrombin receptor-activating peptide (TRAP, SFLLRN-NH2) was from Bachem (Torrance, CA). Adenosine 5′-diphosphate (ADP) was from Chrono-log Corporation (Havertown, PA). Vacutainer® 3.2% sodium citrate blood collection tubes were from BD Biosciences (San Jose, CA). HEPES-Tyrode’s buffer with 0.35% bovine serum albumin (HT-BSA; henceforth known as vehicle) (10 mM HEPES, 137 mM sodium chloride, 2.8 mM potassium chloride, 1 mM magnesium chloride, 12 mM sodium hydrogen carbonate, 0.4 mM sodium phosphate dibasic, 5.5 mM glucose, and 0.35% w/v bovine serum albumin, pH 7.4) was made with reagents from Sigma Aldrich (St. Louis, MO). All other chemicals or reagents were from Sigma Aldrich.

### Human blood collection

Donors provided written informed consent in accordance with the Declaration of Helsinki prior to participation in this Boston Children’s Hospital IRB-approved study. Blood was collected by venipuncture with a 21-gauge butterfly needle into evacuated tubes containing 3.2% sodium citrate. Blood was drawn from healthy volunteers or GT patients who were free from antiplatelet agents and non-steroidal anti-inflammatory drugs for 10 days prior to the donation. The blood draws were performed by the same phlebotomist. Complete blood cell counts were performed in a Sysmex XN-1000 Hematology Analyzer.

### Antibody conjugation

Anti-CD29, anti-CD42a, anti-GPVI, and anti-activated αIIbβ3 (PAC1) were conjugated to chelating polymers loaded with lanthanide metals (176Yb, 155Gd, 152Sm and 159Tb, respectively) using a MaxPAR X8 Antibody Labeling Kit and Fluidigm buffers (Buffer L, R, and W) according to the manufacturer’s protocol. The supplied chelating polymer was loaded with the lanthanide metal of choice by co-incubation in Buffer L at 37 °C for 30–40 minutes. Separately, the antibodies were partially reduced in Buffer R solution plus 4 mM TCEP bond breaker solution at 37 °C for 30 minutes and then purified by buffer exchange using a 50 kDa Amicon filter. The metal-loaded polymers were concentrated in a 3 kDa Amicon filter, added to the reduced antibody, and incubated at 37 °C for 1–2 hours for conjugation to occur. Conjugated antibodies were washed free of unreacted polymer and metal ions using Buffer W, quantified by measuring absorbance at 280 nm on a NanoDrop 2000 Spectrophotometer (ThermoFisher Scientific, Waltham, MA), resuspended at a concentration of 0.5 mg/mL in Antibody Stabilization Buffer, supplemented with 0.05% sodium azide and stored long term at 4 °C. Each antibody was titrated to optimal staining concentrations using healthy donor platelets.

### MC analysis of platelets

A panel of metal-labeled antibodies directed against platelet antigens of interest was assembled (Fig. 1A). Antibody clones were well-characterized, widely used and purchased from reputable vendors. Platelets in whole blood were reacted with the panel (containing anti-CD9-171Yb, anti-CD29-176Yb, anti-CD31-145Nd, anti-CD36-150Nd, anti-CD42a-155Gd, anti-CD42b-163Dy, anti-CD41-149Sm, anti-CD62P-172Yb, anti-CD61-165Ho, anti-CD63-161Dy, anti-CD107a-166Er, anti-CD154-154Sm, anti-GPVI-152Sm and anti-PAC1-159Tb; see Fig. 1A, Materials, and Supplemental Table S1 for antibody information) in the presence of vehicle (HT-BSA), TRAP or ADP at the indicated concentrations for 30 minutes (Fig. 1B). Samples were fixed in 1% formaldehyde/HEPES-saline solution containing 125 nM Iridium 191/193 Cell-ID DNA Intercalator for 30 minutes. Cells were washed two times in MilliQ deionized H_2_O to remove salts and resuspended in 0.5 mL of MilliQ deionized H_2_0 containing EQ Four Element Calibration Beads (1:10 v/v [~33,000 beads/mL]). Samples were passed through a 35 µm cell-strainer (Corning Inc, Corning, NY) and analyzed on a Helios Mass Cytometer (Fluidigm Corporation, San Francisco, CA; Fig. 1C). Cell events were acquired at 300–500 events per second and ≥30,000 events were acquired in total. Platelets were gated based on DNA content (DNA-low) and CD41/CD61 expression (see Supplemental Figure S3 for the MC platelet gating strategy). High-dimensional analyses of platelet subpopulations were carried out using the visual stochastic neighbor embedding (viSNE) cluster analysis function in Cytobank™ software (www.cytobank.org)^67^. Experiments were carried out by the same scientist and all reagents were from the same lot.

### FFC analysis of platelets

Whole blood flow cytometric analysis of platelet activation was performed as previously described^45,68,69^. Three color analysis was performed using two cocktails of fluorescently labeled antibodies: PE-conjugated anti-CD62P, FITC-conjugated PAC1 (directed against the high affinity conformation of integrin αIIbβ3) and PE-Cy5-conjugated anti-CD42b; or PE-conjugated anti-CD41a, FITC-conjugated anti-CD61 and PE-Cy5-conjugated anti-CD42b (see Materials and Supplemental Table S2 for antibody information). Citrate-anticoagulated whole blood was treated with vehicle, TRAP or ADP at the indicated concentrations in the presence of the appropriate fluorescently labeled antibody cocktail for 30 minutes at ambient temperature. Samples were fixed in 1% formaldehyde/HEPES-saline buffer for 30 minutes prior to analysis in a FACSCalibur flow cytometer (BD Biosciences, San Jose, CA). Platelets were gated based on forward light scatter, side light scatter and CD42b expression (see Supplemental Figure S3 for FFC platelet gating strategy). A total of 15,000 platelet events per sample were collected. Experiments were carried out by the same scientist and all reagents were from the same lot.

### Statistical analysis

Data were analyzed using GraphPad version 5.0 software (GraphPad Software, La Jolla, CA) and are presented as mean ± standard error of the mean. An extra sum-of-squares *F* test was performed to determine differences in EC_50_ values between dose-response curves constructed using FFC and MC. Data used for statistical analysis was tested using a 1-way ANOVA in conjunction with a Dunnett multiple comparison test/Bonferroni post-test or a 2-way ANOVA with a Bonferroni post-test.

### Data availability

The datasets generated during and/or analyzed during the current study are available from the corresponding author on reasonable request.

## Electronic supplementary material

Supplementary Information
