# Supplementary material for: Mass Cytometry Reveals Distinct Platelet Subtypes in Healthy Subjects and Novel Alterations in Surface Glycoproteins in Glanzmann Thrombasthenia
Source: Sci Rep. 2018 Jul 9;8:10300. doi: 10.1038/s41598-018-28211-5 (PMC6037710; doi:10.1038/s41598-018-28211-5)
Supplement: Supplementary file 1 — Supplementary Information [file 41598_2018_28211_MOESM1_ESM.pdf]

Mass Cytometry Reveals Distinct Platelet Subtypes in Healthy Subjects and Novel Alterations in Surface Glycoproteins in Glanzmann Thrombasthenia

Thomas A. Blair, Alan D. Michelson and Andrew L. Frelinger III
Center for Platelet Research Studies, Dana-Farber/Boston Children's Cancer and Blood Disorders Center, Harvard Medical School, Boston, MA

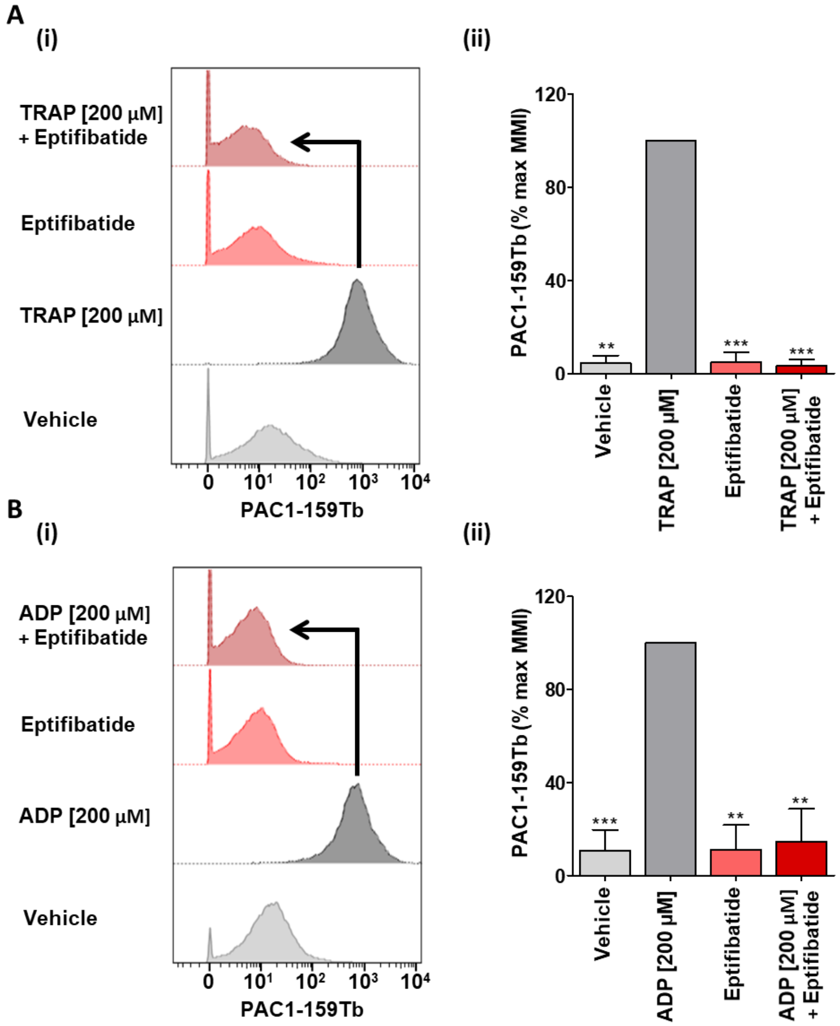

Supplemental Figure S1. Measuring the specificity of in-house metal-tagged PAC1 for integrin αIIbβ3. (A-B) Citrate-anticoagulated blood was treated with 200 μM TRAP/ADP, 3.33 μg/mL eptifibatide or TRAP/ADP plus eptifibatide in combination for 30 minutes in the presence of PAC-1-159Tb. Samples were fixed in 1% formaldehyde and analyzed by mass cytometry. Representative histograms demonstrating the mean metal intensity (MMI) are displayed **(A(i), B(i))** along with bar charts with results expressed as a percentage of the MMI achieved with 200 μM TRAP/ADP (mean ± SEM; n=3 **(A(ii), B(ii))**). Statistical analysis: 1-way ANOVA was used in conjunction with a Bonferroni post-test (with all results compared to the MMI achieved with agonist stimulation) to indicate statistical significance; **P<0.01 and ***P<0.001. Abbreviations: ADP, adenosine diphosphate; TRAP, thrombin receptor activating peptide.

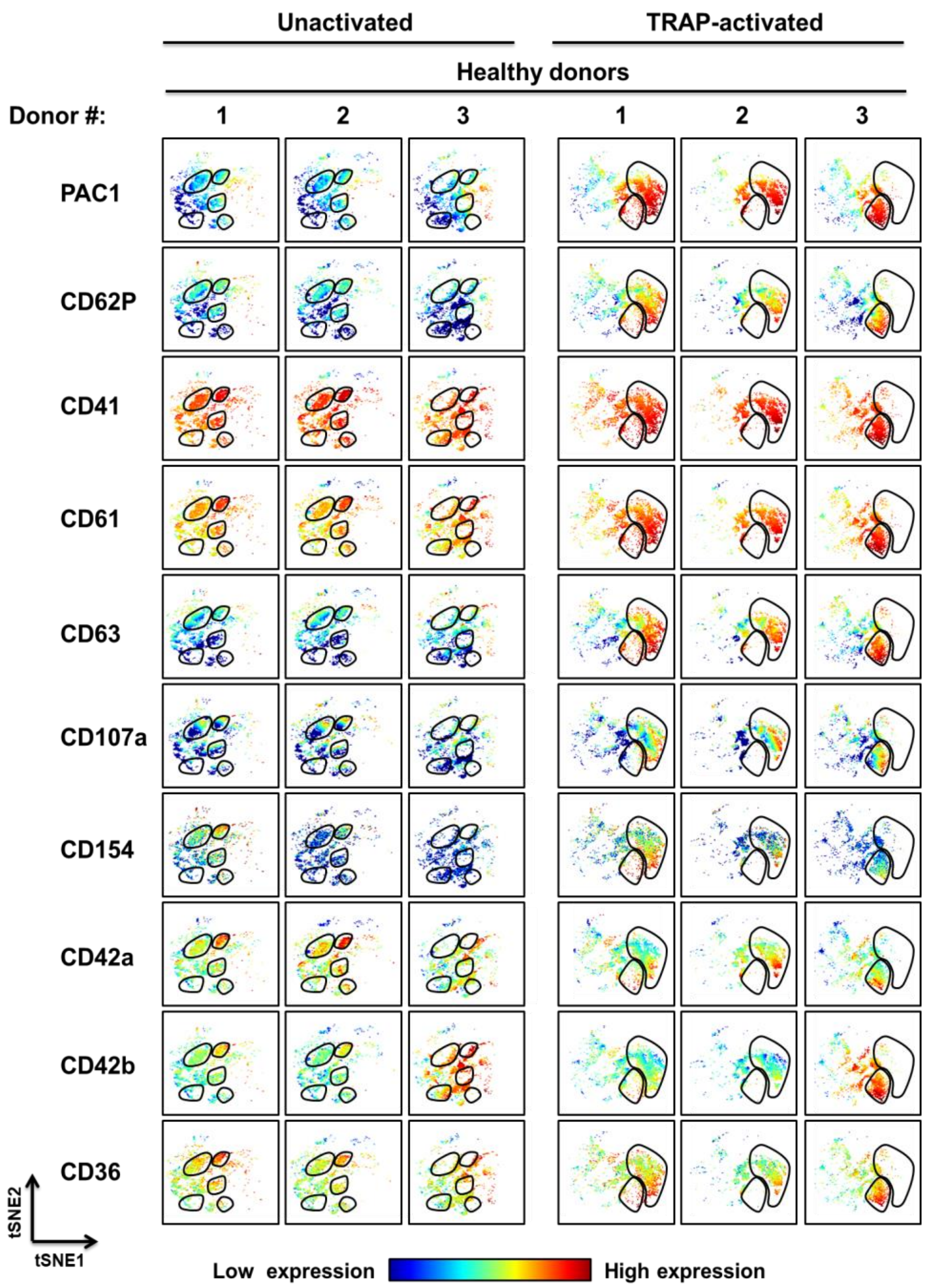

Supplemental Figure S2. Multidimensional analysis of platelets by MC reveals common and private platelet subpopulations in 3 different healthy donor samples. Visual stochastic neighbor embedding (viSNE) plots of whole blood samples drawn from 3 separate healthy donors. Samples were stained with a metal-tagged antibody cocktail containing 10 markers (directed against: CD36, CD41, CD42a, CD42b CD61, CD63, CD62P, CD107a, CD154 and PAC1), treated with vehicle of 20 µM TRAP, and analyzed using MC. Color intensity relates to antigen expression (low [blue] or high [red]) and each dot represents an individual platelet. The distance between dots/platelets and populations of dots/platelets is inversely proportional to how closely related those dots/platelets are in terms of antigen expression and characteristics. Abbreviations: TRAP, thrombin receptor activating peptide; tSNE, t-distributed stochastic neighbor embedding.

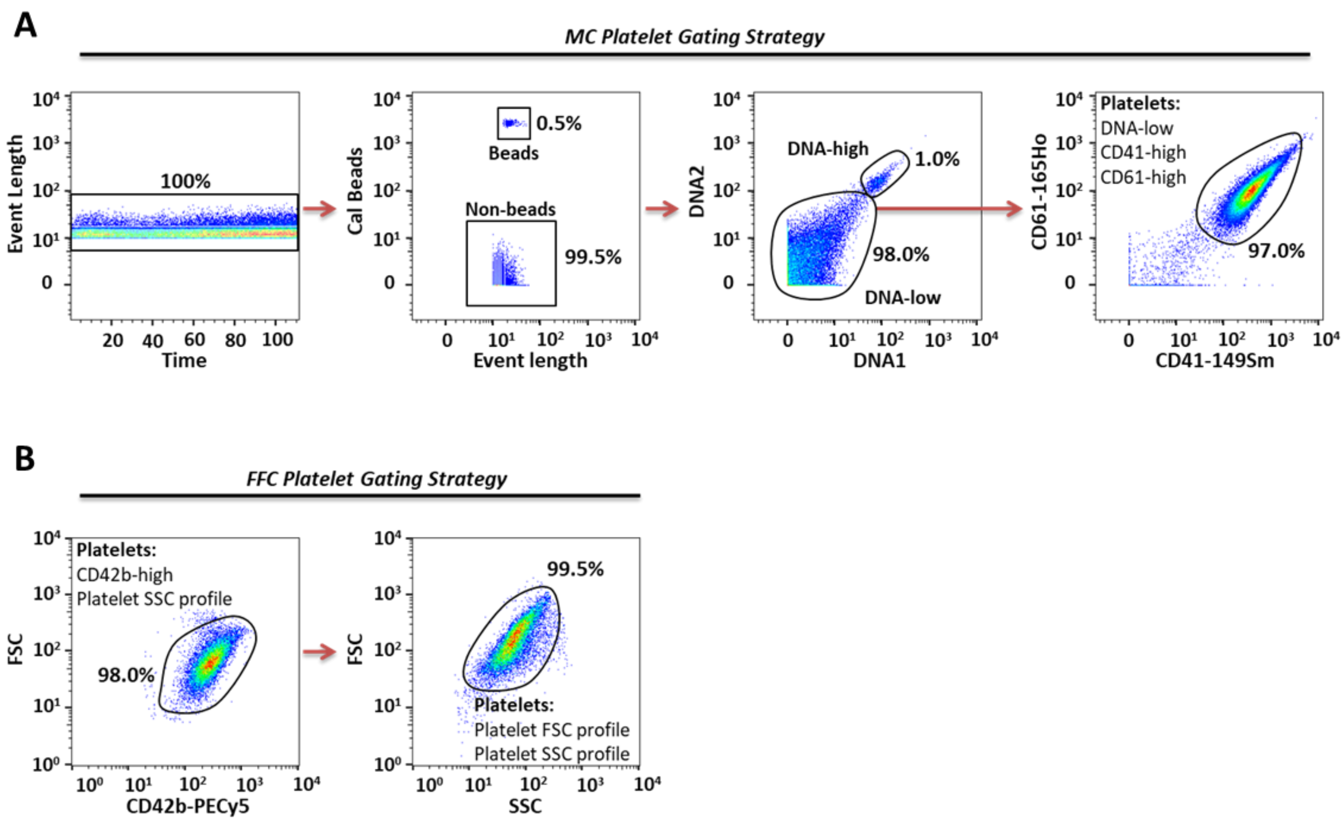

Supplemental Figure S3. Platelet gating strategy for MC and FFC. Platelets are identified as DNA-low and CD41/CD61-high by MC **(A)**. For Glanzmann thrombasthenia studies platelets are identified as DNA-low and CD42a/CD42b-high by MC. Platelets are identified by typical forward- and side-scatter properties and CD42b-high by FFC **(B)**. Abbreviations: DNA, deoxyribonucleic acid; FFC, fluorescence flow cytometry; FSC, forward-scatter MC, mass cytometry; SSC, side-scatter.

Antigen	Common name	Clone	Antibody type	Metal tag	Tag type	Manufacturer	Final conc. (µg/mL)
CD9	Tetraspanin	SN4 C33A2	IgG; monoclonal	171Yb	C	Fluidigm	5
CD29	Integrin β1	TS2/16	IgG; monoclonal	176Yb	I	Biolegend	5
CD31	PECAM-1	WM59	IgG; monoclonal	145Nd	C	Fluidigm	5
CD36	GPIV	5-271	IgG; monoclonal	150Nd	C	LMAAC	2
CD42a	GPIX	ALMA.16	IgG; monoclonal	155Gd	I	BD Biosciences	2.5
CD42b	GPIbα	HIP1	IgG; monoclonal	163Dy	C	LMAAC	3.5
CD41	Integrin αIIb	HIP8	IgG; monoclonal	149Sm	C	LMAAC	2
CD62P	P-selectin	AK4	IgG; monoclonal	172Yb	C	LMAAC	3.5
CD61	Integrin β3	VI-PL2	IgG; monoclonal	165Ho	C	Fluidigm	5
CD63	LAMP-3	H5C6	IgG; monoclonal	161Dy	C	LMAAC	3.5
CD107a	LAMP-1	H4A3	IgG; monoclonal	166Er	C	LMAAC	3.5
CD154	CD40L	24-31	IgG; monoclonal	154Sm	C	LMAAC	3.5
GPVI	GPVI	N/A	IgG; polyclonal	152Sm	I	EMD Millipore	7.5
Activated αIIbβ3	Activated αIIbβ3	PAC-1	IgM, monoclonal	159Tb	I	BD Biosciences	7.5-17

Supplemental Table S1. A list of metal-tagged antibodies used for MC experiments.
Abbreviations: C, commercial; CD, cluster of differentiation; GP, glycoprotein; I, in-house; Ig, immunoglobulin; LMAAC, Longwood Medical Area Antibody Core; MC, mass cytometry.

Antigen	Common name	Clone	Antibody type	Fluorescent tag	Tag type	Manufacturer	Final conc. (μg/mL)
CD41a	Integrin αIIb	HIP8	IgG, monoclonal	PE	C	BD Biosciences	1:15 final dilution of stock
CD42b	GPIbα	HIP1	IgG, monoclonal	PE-Cy5	C	BD Biosciences	1
CD61	Integrin β3	Y2/51	IgG, monoclonal	FITC	C	Agilent	1:125 final dilution of stock
CD62P	P-selectin	AK4	IgG, monoclonal	PE	C	BD Biosciences	1.5
Activated αIIbβ3	Activated αIIbβ3	PAC1	IgM, monoclonal	FITC	C	BD Biosciences	40

Supplemental Table S2. A list of fluorescent-tagged antibodies used for FFC experiments.
Abbreviations: C, commercial; CD, cluster of differentiation; Cy, cyanine; FFC, fluorescence flow cytometry; FITC, fluorescein isothiocyanate; GP, glycoprotein; Ig, immunoglobulin; PE, phycoerythrin.
